# Self‐Emulsifying Drug Delivery Systems (SEDDS) Containing Reverse Micelles: Advanced Oral Formulations for Therapeutic Peptides

**DOI:** 10.1002/adhm.202302034

**Published:** 2023-09-20

**Authors:** Arne Matteo Jörgensen, Christian Steinbring, Daniel Stengel, Dennis To, Pascal Schmid, Andreas Bernkop‐Schnürch

**Affiliations:** ^1^ Department of Pharmaceutical Technology University of Innsbruck Institute of Pharmacy Center for Chemistry and Biomedicine Innrain 80–82 Innsbruck 6020 Austria

**Keywords:** drug release, nanocarriers, oral peptide delivery, payload enhancement, polymyxin B

## Abstract

Alternative methods to hydrophobic ion pairing for the formation of lipophilic complexes of peptide drugs to incorporate them in lipid‐based nanocarriers such as self‐emulsifying drug delivery systems (SEDDS) for oral administration are highly on demand. Such an alternative might be reverse micelles. Within this study, SEDDS containing dry reverse micelles (dRMs_PMB_) formed with an anionic (sodium docusate; AOT), cationic (dimethyl‐dioctadecyl‐ammonium bromide; DODAB), amphoteric (soy lecithin; SL), or non‐ionic (polysorbate 85; P85) surfactant loaded with the model peptide drug polymyxin B (PMB) are developed. They are characterized regarding size, payload, release kinetics, cellular uptake, and peptide activity.

SEDDS exhibit sizes from 22.2 ± 1.7 (AOT‐SEDDS‐dRMs_PMB_) to 61.7 ± 3.2 nm (P85‐SEDDS‐dRMs_PMB_) with payloads up to 2% that are approximately sevenfold higher than those obtained via hydrophobic ion pairing. Within 6 h P85‐SEDDS‐dRMs_PMB_ and AOT‐SEDDS‐dRMs_PMB_ show no release of PMB in aqueous medium, whereas DODAB‐SEDDS‐dRMs_PMB_ and SL‐SEDDS‐dRMs_PMB_ show a sustained release. DODAB‐SEDDS‐dRMs_PMB_ improves uptake by Caco‐2 cells most efficiently reaching even ≈100% within 4 h followed by AOT‐SEDDS‐dRMs_PMB_ with ≈20% and P85‐/SL‐SEDDS‐dRMs_PMB_ with ≈5%. The peptide drug maintains its antimicrobial activity in all SEDDS‐dRMs_PMB_.

According to these results, SEDDS containing dRMs might be a game changing strategy for oral peptide drug delivery.

## Graphical abstract

Self‐emulsifying drug delivery systems containing “dry” reverse micelles (SEDDS‐dRMs) formed with an anionic (AOT), cationic (DODAB), amphoteric (SL), or non‐ionic (P85) surfactant loaded with the model peptide drug polymyxin B (PMB) are developed. Resulting SEDDS‐dRMs_PMB_ demonstrate promising characteristics regarding payload, cellular interaction as well as peptide activity and thus might represent a game changing strategy for oral peptide drug delivery.

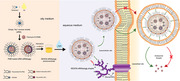

## 1 Introduction

For decades, therapeutic peptides have experienced an ongoing success story in which their oral administration is favored from a patient and commercial point of view.^[^ ^1^ ^]^ Oral administration of peptide drugs, however, bears numerous risks, including inactivation under harsh pH conditions, enzymatic degradation by gastrointestinal (GI) peptidases, thiol/disulfide exchange reactions with endogenous thiols and poor membrane permeability.^[^ ^2^ ^]^ Their size and solubility profile additionally raise challenges for the development of suitable carrier systems.

Among numerous strategies for oral peptide delivery, lipid‐based nanocarriers turned out to be one of the most promising.^[^ ^1^ ^]^ In particular, self‐emulsifying drug delivery systems (SEDDS) showed encouraging results with a comparatively high oral bioavailability of various peptide drugs.^[^ ^3^ ^]^

By the formation of hydrophobic ion pairs (HIPs) even highly hydrophilic peptides can be incorporated in the lipophilic phase of these nanocarriers.^[^ ^4^ ^]^ Although the distribution coefficient (logD) of numerous peptide drugs can be tremendously raised by the formation of HIPs, this method has a considerable shortcoming as it strictly depends on the number of charged groups on the therapeutic agent. If a peptide bears just a few anionic or cationic moieties, its lipophilicity cannot be raised sufficiently via HIP formation. As a consequence, just low payloads are obtained and HIPs are rapidly released from the lipophilic carrier system before they have reached the absorption membrane.^[^ ^3^, ^5^ ^]^ Alternative methods for the formation of lipophilic complexes of peptide drugs are thus highly on demand. Such an alternative method might be the formation of reverse micelles (RMs). They consist of an external shell formed by the hydrocarbon chains of surfactants with internally directed polar charged or uncharged head‐groups. Hydrophilic drugs can serve as polar counterparts localized in the interior of these colloids providing a unique opportunity to solubilize them in the oily phase.^[^ ^6^ ^]^ Generally, RMs can be divided into “wet” and “dry” systems.^[^ ^7^ ^]^ The aqueous or solvent containing core of “wet” RMs enables the incorporation of high amounts of hydrophilic drugs. In contrast, it is more challenging to incorporate them in “dry” RMs since water or other hydrophilic solvents are missing. Nonetheless, “dry” RMs (dRMs) are preferred over “wet” RMs, as storage stability problems of peptide drugs such as hydrolytic cleavages can be avoided and lipophilic complexes of drugs and surfactants exhibit a comparatively higher membrane permeability.^[^ ^8^ ^]^ So far, however, the potential of dRMs has not be utilized for the incorporation of peptide drugs in SEDDS. It was therefore the aim of this study to develop and characterize SEDDS containing dRMs for oral peptide drug delivery.

To that end, dRMs were formed with non‐ionic, anionic, cationic, and amphoteric surfactants and the model peptide drug polymyxin B (PMB) was incorporated. Entrapment efficiency (EE) and logD of the formed complexes were determined. Subsequently, SEDDS containing PMB loaded dRMs (SEDDS‐dRMs_PMB_) were developed. SEDDS‐dRMs_PMB_ were characterized regarding size, polydispersity index (PDI), ζ‐potential, drug loading capacity, self‐emulsification time, drug release and cytotoxicity. Cellular uptake and endosomal escape of these SEDDS were evaluated. The antimicrobial activity of PMB in SEDDS‐dRMs_PMB_ was determined against *Escherichia coli (E. coli)*.

## 2 Results and Discussion

### 2.1 Development and Characterization of Dry Reverse Micelles

**Figure 1:**
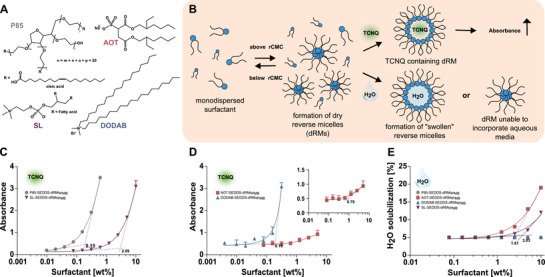
A) Surfactants used for dRMs – non‐ionic: P85, anionic: AOT, cationic: DODAB and amphoteric: SL. B) Mechanism of rCMC determination via TCNQ‐ and water‐solubilization method. C) Plots of absorbance of TCNQ solubilized in oil containing P85 (grey ●) and SL (violet ▼) at a wavelength of 480 nm and D) of DODAB (blue ▲) and AOT (red ■) at a wavelength of 850 nm as a function of logarithm of surfactant concentration [wt%]. E) Plots of water solubilization as a function of logarithm of surfactant conc. [wt%]. The numbers at intersection points indicate the individual rCMC. Data are shown as means ± SD (*n* ≥ 3).

The dRMs were formed by dissolving surfactants (**Figure** **1**A) in an oily phase that can subsequently also be used for the formation of SEDDS. The reverse critical micellar concentration (rCMCs) was determined to be 0.15% for polysorbate 85 (P85), 0.15% for dimethyl‐dioctadecyl‐ammonium bromide (DODAB), 0.79% for sodium docusate (Aerosol OT; AOT) and 2.09% for soy lecithin (SL) by 7,7,8,8‐tetracyanoquinomethane (TCNQ)‐solubilization method (Figure 1C,D).

The water‐solubilization method showed rCMCs of 1.81% for AOT and 3.03% for SL that are roughly in line with rCMCs determined by TCNQ method (Figure 1E). These results are in agreement with numerous other reverse micellar systems solubilizing water by forming water‐pools in their interior as illustrated in Figure 1B.^[^ ^9^, ^10^ ^]^ Since the water solubilization capacity of oily solutions containing P85 or DODAB did not increase, even at the highest tested concentrations that are above their rCMC, however, water uptake of these micelles could be excluded.

Although numerous hydrophilic drugs can be dissolved at high concentrations in the aqueous inner phase of w/o emulsions, the uptake of aqueous media into dRMs is in case of oral peptide delivery not advantageous at all. After oral administration, GI fluids containing peptidases as well as thiols such as glutathione will enter RMs degrading the incorporated peptide drug.^[^ ^1^, ^9^ ^]^ A drop in pH due to the uptake of gastric fluid might also destabilize peptide drugs. Moreover, the uptake of aqueous media will likely enhance undesired extraction of cargo from micellar systems.^[^ ^10c^ ^]^ Therefore, DODAB and P85 are likely superior to AOT and SL forming non‐water binding dRMs.

**Figure 2:**
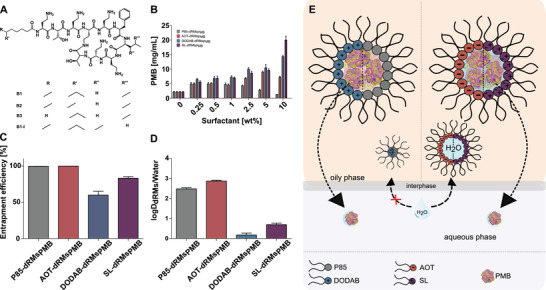
A) Structure of PMB. B) Maximum payloads of PMB in dRMs formed at increasing surfactant concentrations. C) EE of different dRMs_PMB_ and D) logD_dRMs/Water_ after 24 h at 37 °C. Data are shown as means ± SD (*n* ≥ 3). E) Anticipated appearance of dRMs formed by different surfactants in oil and their behavior in a 2‐phase system to determine EE of PMB (not drawn to scale).

PMB (**Figure** **2**A) was incorporated in dRMS in dry form. The maximum amount of PMB that could be incorporated in each type of dRMs is shown in Figure 2B.

For AOT, the maximum PMB payload of 9.02 ± 0.31 mg mL^−1^ was reached at a concentration of 5% (Figure 2B). In case of P85, the highest payload of 4.91 ± 0.56 mg mL^−1^ was achieved at a concentration of 0.25%. Above this concentration drug solubility decreased and at a concentration of 10% it dropped even below the solubility of PMB in the pure oily phase.

In contrast, the higher the concentration of DODAB was, the higher was the payload of PMB reaching a maximum of 14.33 ± 0.37 mg mL^−1^ at a concentration of 10%. SL‐dRMs showed a similar solubilization profile with a moderately enhanced solubility of 9.60 ± 0.55 mg mL^−1^ at 5% surfactant concentration. The PMB concentration nearly doubled by reaching a payload of 20.01 ± 1.35 mg mL^−1^ at 10% surfactant concentration. This payload was even almost tenfold higher than that determined in the oily phase without dRMs. The more surfactant is added, the higher is the diameter of dRMs and the easier the peptide can be incorporated.^[^ ^11^ ^]^ In dRMs containing the non‐ionic surfactant P85 solvation of PMB is likely related to intermolecular hydrogen‐bonds,^[^ ^12^ ^]^ whereas in case of all other surfactants additional ionic interactions seem to be involved (Figure 2E).^[^ ^9^ ^]^ Although, PMB and DODAB are both positively charged, DODAB‐based dRMs provided higher PMB solubility than AOT‐dRMS. The sulfate counterion of PMB might bind the drug and cationic surfactant at the same time as it is known for calcium ions binding two fatty acids at the same time.

According to these results, the most promising dRMs_PMB_ were formed by 0.25% P85, 5% AOT, 10% DODAB and 10% SL. These dRMs_PMB_ were chosen for further investigations. EE of PMB in P85‐ and AOT‐dRMs_PMB_ was 100%, whereas it was ≥ 55% and ≥ 81% for DODAB‐ and SL‐dRMs_PMB_, respectively (Figure 2C). In agreement with these results, the calculated logD of PMB characterizing its distribution between dRMs and water was for P85 and AOT containing dRMs higher than for those of DODAB‐ and SL‐ based dRMs_PMB_ (Figure 2D).

These results might be explained by non‐ionic and ionic interactions between PMB and the surfactants illustrated in Figure 2E. In case of P85 and AOT, non‐ionic and ionic interactions between PMB and the surfactant were likely responsible for an EE of 100%, respectively. In contrast, the formation of rather unstable complexes between PMB, sulfate and surfactant and the zwitterionic character of SL might explain the lower EE of DODAB‐dRMs_PMB_ and SL‐dRMs_PMB_, respectively.

### 2.2 Development and Characterization of Self‐Emulsifying Drug Delivery Systems Containing dRMs_PMB_

**Figure 3:**
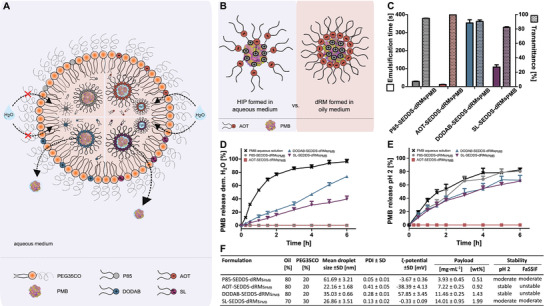
A) Anticipated appearance of different types of SEDDS‐dRMs emulsified in water (not drawn to scale). B) HIP formation in aqueous media versus dRMs in oily solution. C) Self‐emulsification‐time (plain column) and transmittance (dotted column) of SEDDS‐dRMs_PMB_ in demineralized water at 37 °C. D/E) Cumulative release of PMB from P85‐ (grey ●), AOT (red ■), DODAB (blue ▲), and SL‐SEDDS‐dRMs_PMB_ (violet ▼) in contrast to the control (black x) emulsified in D) demineralized water (1:10) and E) in 0.01 m HCl (pH 2) within 6 h of incubation at 37 °C. F) Droplet size [nm], PDI, ζ‐potential [mV] of SEDDS diluted in demineralized water 1:100 at 37 °C, their payload and stability in biorelevant media. Data are shown as means ± SD (*n* ≥ 3).

For the development of SEDDS‐dRMs_PMB_, the concentration of the emulsifier PEG35‐castor oil (PEG35CO) was kept as low as feasible, since potential toxic effects are associated with high emulsifier concentrations. Based on orientating studies, 20% (v/v) of PEG35CO were sufficient to provide self‐emulsifying properties for dRMs_PMB_. Merely in case of dRMs_PMB_ based on SL, the concentration of PEG35CO had to be raised up to 30% in order to achieve sufficient self‐emulsification. The anticipated appearance of SEDDS‐dRMs_PMB_ is illustrated in **Figure** **3**A.

The mean droplet size ranged from 61.7 ± 3.2 (P85‐SEDDS‐dRMs_PMB_) to 22.2 ± 1.7 nm (AOT‐SEDDS‐dRMs_PMB_) with a PDI ≤ 0.4 (Figure 3F). The comparatively high PDI of AOT‐SEDDS‐dRMs_PMB_ can be explained by the formation of micelles due to the release of water‐soluble AOT from SEDDS. These micelles occur as another population with a size of ≈10 nm in the histogram of the size distribution by intensity obtained by DLS (Figure S1, Supporting Information).

The ζ‐potential depended strongly on the type of surfactant showing that these auxiliary agents do not just remain within dRMs but assemble also on the surface of oily droplets. In case of non‐ionic P85‐ and amphoteric SL‐based SEDDS‐dRMs_PMB_, ζ‐potential was close to zero. Anionic AOT‐SEDDS‐dRMs_PMB_ exhibited a pronounced negative ζ‐potential and cationic DODAB‐SEDDS‐dRMs_PMB_ were highly positively charged.

P85‐SEDDS‐dRMs_PMB_ showed the lowest payload with 0.51%, whereas SEDDS comprising SL provided a fourfold higher PMB payload with 1.99%. Overall, SEDDS‐dRMs_PMB_ can be ranked in the following order with increasing payload: P85‐SEDDS‐dRMs_PMB_ < AOT‐SEDDS‐dRMs_PMB_ < DODAB‐SEDDS‐dRMs_PMB_ < SL‐SEDDS‐dRMs_PMB_. Results are encouraging as these payloads were higher than those having been achieved with hydrophobic ion pairs (HIPs). In fact, a maximum payload of 5 mg·mL^−1^ PMB HIPs formed at a molar ratio of 1:2 with agaric acid that corresponds to 2.94 mg mL^−1^ of pure PMB or a payload of just 0.29% could so far be reached.^[^ ^13^ ^]^ In case of hydrophobic ion pairing, just eight hydrophobic anionic counterions can be bound to the amine substructures of PMB, whereas in case of dRMs all hydrophilic substructures on the peptide are shielded by surfactant molecules (Figure 1B). The consequently higher lipophilic character of these complexes results in a higher solubility in the oily phase and a higher payload.

The self‐emulsifying properties of SEDDS were dependent on their composition (Figure 3C). AOT‐SEDDS‐dRMs_PMB_ showed the shortest emulsification time of 11.5 ± 1.2 s, followed by P85‐SEDDS‐dRMs_PMB_ with 28.5 ± 1.4 s, SL‐SEDDS‐dRMs_PMB_ with 108.3 ± 12.3 s and DODAB‐SEDDS‐dRMs_PMB_ with 354 ± 17.7 s for complete emulsification. These results correlate directly with the viscosity of preconcentrates (Figure S3, Supporting Information) and are consistent with the literature. It is reported that the viscosity of formulation is a key parameter for their self‐emulsification.^[^ ^14^ ^]^ A high preconcentrate viscosity restricts interfacial surface shearing and water penetration decelerating consequently the self‐emulsification process.^[^ ^15^ ^]^ Thus, the higher preconcentrate viscosity of DODAB‐SEDDS‐dRMs_PMB_ is likely responsible for the extended self‐emulsification time. Drug loading did not seem to affect the emulsification time of SEDDS‐dRMs_PMB_, since SL‐SEDDS‐dRMs_PMB_ containing the highest amount of PMB emulsified faster than DODAB‐SEDDS‐dRMs_PMB_. Similarly, AOT‐SEDDS‐dRMs_PMB_ emulsified more rapid than P85‐SEDDS‐dRMs_PMB_, although containing a lower PMB payload. Consequently, the surfactants used to form dRMs play the key role for emulsification time. In order to improve self‐emulsification time, co‐surfactants or co‐solvents might be applied.^[^ ^16^, ^17^ ^]^

In biorelevant media, AOT‐SEDDS‐dRMs_PMB_ and DODAB ‐SEDDS‐dRMs_PMB_ showed satisfying stability at pH 2 by providing stable droplet size of 42.67 ± 2.54 and 56.72 ± 5.4 nm, respectively, with narrow PDIs of 0.07 ± 0.03 and 0.21 ± 0.01 on average over 24 h. In contrast, P85‐SEDDS‐dRMs_PMB_ and SL‐SEDDS‐dRMs_PMB_ started to swell ≈3.5‐ and ≈2‐fold, respectively, with increasing PDIs that were still indicating monodisperse systems for the former but for the latter solely during 4 h of incubation time.

In FaSSIF (fasted simulated intestinal fluid) on contrary, all SEDDS were prone to interactions with bile salts or lipids comprising the simulated intestinal fluid. These interactions resulted in more pronounced changes in size (approximately eightfold for P85‐SEDDS‐dRMs_PMB_ and almost sevenfold for SL‐ P85‐SEDDS‐dRMs_PMB_) or instability. P85‐SEDDS‐dRMs_PMB_ and SL‐ P85‐SEDDS‐dRMs_PMB_ provided monodisperse systems (PDI < 0.24) up to 4 h under these conditions suggesting moderate stability.

### 2.3 Drug Release Studies

Results of LogD_dRMs/RM_ determinations (Figure 2D) were additionally confirmed by drug release studies using a semipermeable membrane. In demineralized water, PMB release from P85‐SEDDS‐dRMs_PMB_ and AOT‐SEDDS‐dRMs_PMB_ remained below the limit of detection throughout the experiment. Since P85 presumably forms stable hydrogen bonds with PMB and AOT presumably forms stable ion pairs with the counterion PMB, the drug is not released from oily droplets of SEDDS (Figure 3E). In contrast, DODAB‐SEDDS‐dRMs_PMB_ showed an almost zero order release kinetic, reaching 73.64% drug release after 6 h. SEDDS containing SL‐dRMs_PMB_ displayed an even slower almost zero order release kinetic with 39.46% of released PMB after 6 h. All SEDDS‐dRMs_PMB_ showed either no or a sustained drug release, compared to the aqueous solution. These results are in agreement with EE data of dRMs_PMB_ shown in section 2.1. By simulating the harsh GI environment (pH 2), drug release from DODAB‐SEDDS‐dRMs_PMB_ and SL‐SEDDS‐dRMs_PMB_ was found to be faster and more extensive. Even P85‐SEDDS‐dRMs_PMB_ showed a rapid and complete drug release which did not occur under neutral conditions. This might be explained by the reduced stability of P85‐SEDDS‐dRMs_PMB_ and SL‐SEDDS‐dRMs_PMB_ (Figure 3F) as well as by the destabilized binding of PMB in the interior of DODAB‐SEDDS‐dRMs_PMB_. Since this binding and consequently the successful entrapment of PMB in DODAB‐SEDDS‐dRMs_PMB_ is provided by the sulfate counterions, it is less stable than the direct ion pairing with negatively charged AOT in AOT‐SEDDS‐dRMs_PMB_ that showed no release at all.

### 2.4 Cytotoxicity Studies

**Figure 4:**
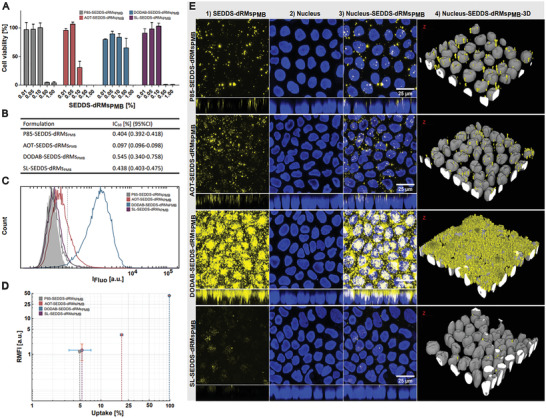
A) Cell viability of Caco‐2 cells after incubation with P85‐SEDDS‐dRMs_PMB_ (grey bars), AOT‐SEDDS‐dRMs_PMB_ (red bars), DODAB‐SEDDS‐dRMs_PMB_ (blue bars) and SL‐SEDDS‐dRMs_PMB_ (violet bars) emulsified in HBS and B) IC_50_ values of SEDDS in percent. Data are shown as means ± SD (*n* ≥ 3). C–E) Cellular uptake of dRMs_PMB_ after 4 h incubation on Caco‐2 cell line at a concentration of 0.025% (v/v) by flow cytometry. Lumogen RED (LGR) was used as lipophilic fluorescence marker embedded in SEDDS. C) Shows the shift in fluorescence intensity of P85‐ (grey), AOT‐ (red), DODAB‐ (blue), and SL‐SEDDS‐dRMs_PMB_ (violet) in contrast to the control (grey area; striped line). D) Displays the relative mean fluorescence intensity (RFMI) versus the uptake [%] per formulation. P85‐ (grey bar), AOT‐ (red), DODAB‐ (blue), and SL‐SEDDS‐dRMs_PMB_ (violet). RFMI values show the fluorescent intensity of LGR‐labeled SEDDS per cell. Data are shown as mean ± SD (*n* = 3). E) Cellular uptake visualized by confocal microscopy. P85‐, AOT‐, DODAB‐, and SL‐SEDDS‐dRMs_PMB_ were incubated for 4 h on a Caco‐2 cell line. 1) Fluorescence of LGR incorporated in formulations as fluorescence marker. 2) Nuclei stained with Hoechst 33 528. 3) The 2‐D and 4) 3‐D merged pictures of 1) and 2).

Cytotoxicity of SEDDS‐dRMs_PMB_ was evaluated on Caco‐2 cells. Merely, AOT‐SEDDS‐dRMs_PMB_ caused in a concentration ≥ 0.1% a decrease in cell viability of over 50% as illustrated in **Figure** **4**A. In contrast, minor or no toxicity was observed for all other SEDDS‐dRMs_PMB_ at a concentration of 0.1%. In case of P85‐SEDDS‐dRMs_PMB_ and SL‐SEDDS‐dRMs_PMB_, however, a concentration of 0.5% resulted already in complete cell death, whereas in case of DODAB‐SEDDS‐dRMs_PMB_ cell viability maintained ≈50%. Because of the highly positive ζ‐potential of DODAB‐SEDDS‐dRMs_PMB_ (Figure 3F), this result was unexpected, since cationic nanocarriers are in most cases more toxic than uncharged or anionic ones.^[^ ^18^ ^]^ Whether the use of cationic surfactants in SEDDS below or above their rCMC has an impact on the cytotoxicity of these formulations deserves further investigations. Taking the IC_50_ values into account, the 95% confidence interval (95%CI) indicated similar toxicity of all tested formulations except AOT‐SEDDS‐dRMs_PMB_ (Figure 4B). The higher toxicity of AOT comprising SEDDS‐dRMS_PMB_ might be explained by the water solubility of this surfactant. Since AOT is the only water‐soluble surfactant, it might be to some extent released from the oily droplets into the aqueous phase interacting more intensively with cells.

### 2.5 Cellular Uptake and Intracellular Fate of SEDDS‐dRMs_PMB_

Flow cytometry and live‐cell imaging were employed to assess the impact of surfactant charge on the uptake of SEDDS‐dRMs_PMB_ by Caco‐2 cells. The histogram in Figure 4C displays a prominent shift in fluorescence intensity distribution of DODAB/AOT‐SEDDS‐dRMs_PMB_ and a minor shift of T85/SL‐SEDDS‐dRMs_PMB_ toward higher values in comparison to control, indicating improved cellular uptake. The relative mean fluorescence intensity (RFMI) versus resulting uptake [%] per formulation is illustrated in Figure 4D.

When cationic DODAB‐dRMs_PMB_ were incorporated into SEDDS, SEDDS‐cell interactions increased significantly resulting in a 10‐ to 40‐fold higher RFMI in comparison to non‐ionic P85‐, anionic AOT‐ or amphoteric SL‐SEDDS‐dRMs_PMB_ and an almost 100% cellular uptake. Negatively charged AOT‐SEDDS‐dRMs_PMB_ showed a cellular uptake of ≈20%, whereas cellular uptake of P85 and SL containing formulations was lowest, without a significant difference (*p* < 0.01). Overall, results are in line with the study by Bannunah et al. describing the impact of surface charge on cellular uptake.^[^ ^19^ ^]^ Since surfactants used for the formation of dRMs can be easily substituted by permeation enhancers such as medium chain fatty acids or bile salts additional effects like enhanced cellular uptake or an opening of tight junctions might be introduced in these systems.^[^ ^20^ ^]^

**Figure 5:**
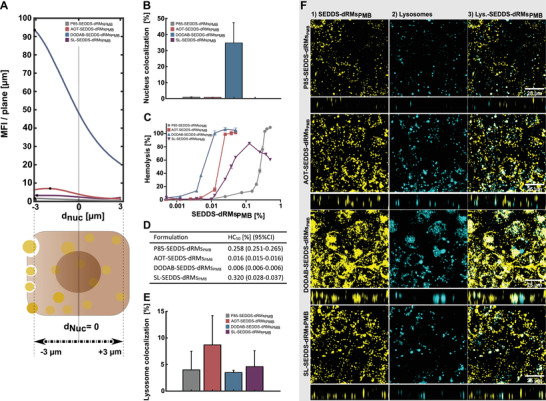
A) Z‐Distribution of SEDDS‐dRMs_PMB_ (mean fluorescence intensity (MFI) per plane) after 4 h incubation on Caco‐2 cell line at a concentration of 0.025%. MFI of P85‐ (grey), AOT (red), DODAB (blue), and SL‐SEDDS‐dRMs_PMB_ (violet) (top). Apparent localization of SEDDS‐dRMs_PMB_ across cell (bottom). B) The colocalization between uptaken SEDDS and the nucleus was determined from recorded image‐data using the ObjectFinder‐application in MatLab. A minimum of a 50% overlap of SEDDS‐objects with the nucleus‐object was counted as colocalized. C) Hemolytic activity in percent of P85‐ (grey), AOT (red), DODAB (blue) and SL‐SEDDS‐dRMs_PMB_ (violet) emulsified in HBS after 4 h incubation at 37 °C and D) HC_50_ values of SEDDS in percent. E) The colocalization between uptaken SEDDS and the lysosomes was determined from recorded image‐data using the ObjectFinder‐application in MatLab. A minimum of a 50% overlap of SEDDS‐objects with a lysosome‐objects was counted as colocalized. F) Cellular uptake visualized by confocal microscopy. P85‐, AOT‐, DODAB‐, and SL‐SEDDS‐dRMs_PMB_ were incubated for 4 h on a Caco‐2 cell line. 1) Fluorescence of Lumogen Orange (LGO) incorporated in formulations as fluorescence marker. 2) Lysosomes stained with LysoView 633. 3) The 2‐D merged pictures of 1) and 2). Data are shown as means ± SD (*n* ≥ 3).

**Figure** **5**A complements the z‐sections in Figure 4E quantitatively. The curves exhibit the MFI/plane along the z‐direction of the cell layer. Each curve represents the average of the MFI/plane within the well. DODAB‐SEDDS‐dRMs_PMB_ showed the highest MFI/plane across the entire cell layer indicating the highest penetration depth and accumulation throughout the cell. The maximum SEDDS‐density for all formulations was measured in the first third of the cell layer (z‐dim), proximal to the cell membrane. Solely for DODAB‐SEDDS‐dRMs_PMB_, a high density was also measured in proximity to the bottom. The data correlate very well with the RMFI‐values, obtained by flow‐cytometry measurements (Figure 4C,D). Furthermore, they provide insights on the cellular distribution of SEDDS‐dRMs_PMB_. According to these results, P85‐, AOT‐ and SL‐SEDDS‐dRMs_PMB_ were predominantly located on top of the cell layer. The results revealed the highest colocalization rate with nucleus for DODAB‐SEDDS‐dRMs_PMB_ (Figure 5B) indicating that this formulation not only enters the cell (Figure 4E) but also the nucleus. In contrast, colocalization with nucleus for other formulations was negligible.

This might be explained by the cationic surface charge of DODAB‐SEDDS‐dRMs_PMB_ and their affinity to the negatively charged cell membrane facilitating the intracellular delivery of the incorporated drugs similar to specially for that purpose designed cationic lipids such as Invitrogen Lipofectamine transfection reagents.^[^ ^21^ ^]^ Cationic lipids form ion pairs with anionic phospholipids of the endosomal membrane after endocytosis, subsequently disrupt the endosomal membrane and promote the release from the endosome to deliver their cargo.^[^ ^22^ ^]^ However, the exact mechanism of the nuclear entry remains unclear.^[^ ^23^ ^]^ One possibility might be a simple diffusion process through nuclear pores.^[^ ^24^ ^]^ Some proteins are known to exhibit nuclear localization signals (NLS) that facilitate their transport into the nucleus. Cationic lipids might interact with these signals aiding in the transport into the nucleus. Other studies suggest an interaction with microtubules that are part of the cytoskeleton and are involved in intracellular transport, DODAB‐SEDDS‐dRMs_PMB_ might hitch a ride on them to reach the vicinity of the nucleus.^[^ ^25^ ^]^ Moreover, direct interactions between the positively charged cationic lipids and the negatively charged components of the nuclear pore might facilitate the transport through the pores. In addition, active transport mechanisms similar to those involved in the import of certain proteins into the nucleus as well as the cellular machinery responsible for DNA repair and recombination might be involved.

Overall, results indicate SEDDS‐dRMs_PMB_ as promising tool for intracellular delivery of PMB broadening its scope in the fight against pathogens. More than two‐thirds of prescribed antibiotics are ineffective against intracellular pathogens as they develop mechanisms against it.^[^ ^26^ ^]^

The endosomal escape properties of nanocarriers corresponds to their hemolytic activity, which is frequently evaluated by assessing the hemoglobin release from RBC ex vivo.^[^ ^27^, ^28^ ^]^ The release is caused by membrane‐carrier interactions and depends on the ability of the carrier system to disrupt the membrane. SEDDS‐dRMS_PMB_ showed substantial differences in membrane interactions in dependence on the surfactant used for dRMs (Figure 5C). P85‐SEDDS‐dRMs_PMB_ had the lowest potential to disrupt the erythrocyte membrane as concentrations > 0.125% were needed to observe a hemolytic effect. The half‐maximal hemolytic activity (HC_50_) was at a concentration of ≈0.258%. AOT‐SEDDS‐dRMs_PMB_ and SL‐SEDDS‐dRMs_PMB_ showed a quite similar onset of hemolysis at ≈0.01% but at higher concentrations, their profiles evolved substantially differently. After having reached a maximum at a concentration of 0.1%, the hemolytic potential of SL‐SEDDS‐dRMs_PMB_ decreased at higher concentrations. This might be attributed to the antihemolytic effect of lecithin,^[^ ^29^ ^]^ that outweighs the membrane disruption potential of the formulation itself at higher SEDDS concentrations. The highest hemolytic activity was observed for DODAB‐SEDDS‐dRMs_PMB,_ exhibiting a HC_50_ of 0.006%. This is in line with the literature as a cationic surface charge causes to a higher extent plasma‐membrane disruption than anionic, amphoteric or non‐ionic‐systems by interacting with negatively charged phospholipid bilayers.^[^ ^30^ ^]^ The endosomal escape properties of SEDDS‐dRMs_PMB_ were further studied by colocalizing each formulation with lysosomal cell organelles (Figure 5F). According to the results, DODAB‐SEDDS‐dRMs_PMB_ exhibited the lowest colocalization rate of < 5% (Figure 5E) preventing the formulation from adverse lysosomal degradation processes and enabling the unhampered transport of cargo within the cytosol. P85‐SEDDS‐dRMs_PMB_ and SL‐SEDDS‐dRMs_PMB_ demonstrated colocalization rates of < 10% and AOT‐SEDDS‐dRMs_PMB_ of < 15% indicating an overall relatively low risk of lysosomal entrapment and thus of degradation.

### 2.6 Antimicrobial Activity of SEDDS‐dRMs_PMB_

**Figure 6:**
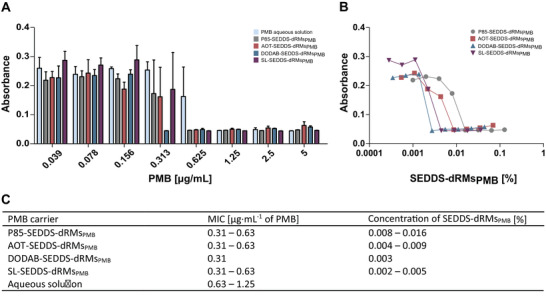
A) Absorbance caused by *E. coli* growth in the presence of indicated concentrations of P85‐ (grey bars), AOT‐ (red bars), DODAB‐ (blue bars), SL‐SEDDS‐dRMs_PMB_ (violet bars) emulsified in sterile water and PMB solution (light blue bars). B) Corresponding concentration of SEDDS‐dRMs_PMB_ in %. C) Resulting MICs of PMB in different formulations. Data are shown as means (*n* ≥ 3) ± SD.

Minimum inhibitory concentration (MIC) was determined to evaluate the antimicrobial activity of PMB when being incorporated in SEDDS‐dRMs. In **Figure** **6**A the absorbance caused by *E. coli* growth was plotted against increasing drug concentrations of indicated formulations.

SEDDS‐dRMs_PMB_ showed MICs ranging from 0.31 to 0.63 µg mL^−1^ against *E. coli* indicating a slightly higher effectivity than unformulated PMB with a MIC of 0.63–1.25 µg mL^−1^ (Figure 6A). SEDDS‐dRMs without PMB showed no antibacterial activity (Table S1, Supporting Information) indicating that the drug having been incorporated in SEDDS‐dRMs is stable and maintains its activity. Although PMB payload in DODAB‐SEDDS‐dRMs_PMB_ was lower than in SL‐SEDDS‐dRMs_PMB_, this formulation showed higher antimicrobial activity (Figure 6B) suggesting a beneficial effect of these SEDDS on the therapeutic efficacy of PMB. The cationic PMB interacts with anionic lipopolysaccharides in the outer membrane and with anionic phospholipids in the inner membrane of Gram‐negative bacteria, leading ultimately to a leakage in bacterial membranes.^[^ ^31^ ^]^ The enhanced activity of SEDDS‐dRMs_PMB_ might be explained by improved membrane permeation of dRMs. Surfactants enhance the binding of drug loaded carriers to the outer bacterial membrane, where the antibiotic drug is released at its target.^[^ ^32^ ^]^ The electrostatic interactions are particularly strong when positively charged delivery systems are used that might explain lower MICs of dRMs formed by DODAB. MIC of PMB having been applied via SEDDS‐dRMs_PMB_ versus aqueous solution as well as the corresponding concentrations of SEDDS are summarized in Figure 6C. According to these results, efficacy of peptide antibiotics might be improved. To date, bacterial infections represent one of the greatest global public health threats of this century with increasing numbers of nosocomial infections and the spread of multidrug‐resistant bacteria.^[^ ^33^ ^]^ The perceived lack of return on investing in the development of new antibacterial drugs has led to a limited number of new antibiotics in the pipeline of the pharmaceutical industry.^[^ ^34^ ^]^ Thus, the improvement of existing antibiotics by developing highly efficient formulations will contribute to our overall strategy to address antibiotic resistance.^[^ ^13^, ^35^ ^]^

## 3 Conclusion

Taken all, this study provides fundamental knowledge for the design of SEDDS containing dRMs. In contrast to hydrophobic ion pairing that can be regarded as the current gold standard for the incorporation of peptide drugs in lipid‐based nanocarriers,^[^ ^1^ ^]^ dRMs allow the attachment of a much higher number of molecules with a lipophilic tail to the surface of these hydrophilic macromolecular drugs. Moreover, these lipophilic complexes can be formed independently from charges that are available on therapeutic peptides. Because of the higher lipophilic character of complexes gained with dRMs essentially higher payloads can be achieved and logD_SEDDS/release medium_ is raised without loss of peptide activity. In particular, DODAB and SL showed promising characteristics in this regard. The provided payloads have been the highest, but so was the drug release in different media that needs to be taken into account. AOT and P85 demonstrated higher EE of cargo in demineralized water, the former even under harsh GI conditions. Additionally, both of them exhibited advantageous faster self‐emulsification compared to DODAB and SL‐based SEDDS‐dRMs_PMB_. In terms of toxicity, cellular uptake, endosomal escape and antimicrobial activity, DODAB‐SEDDS‐dRMs_PMB_ was highly superior among all SEDDS tested and is therefore considered as the overall most promising candidate followed by AOT‐SEDDS‐dRMs_PMB_ and a tie between P85 and SL.

In a broader prospect, this technology might pave the way for oral administration of numerous peptide drugs and even open the door for oral administration of much larger hydrophilic macromolecular drugs such as proteins, nucleotides, polysaccharides and antibodies.

## 4 Experimental Section

### Materials

Tween 85 (polysorbate 85, P85), dimethyl‐dioctadecyl‐ammonium bromide (DODAB), Cremophor EL (PEG35‐castor oil, PEG35CO) trifluoroacetic acid (TFA), 7,7,8,8‐tetracyanoquinodimethane (TCNQ), Triton X‐100 and MüllerHinton‐Broth were all obtained from Sigma‐Aldrich (Steinheim, Germany). Dioctyl sulfosuccinate sodium salt (sodium docusate, Aerosol OT; AOT) was purchased from Alfa Aesar (Kandel, Germany). Soy lecithin Lipoid S 100 (SL) was a gift from Lipoid GmbH (Ludwigshafen, Germany). Miglyol 812 (medium‐chain triglycerides) was supplied by Caelo (Hilden, Germany). Capmul MCM C8 EP/NF (mono‐ and diglycerides of caprylic acid) was a gift from Abitec Corporation (Janesville (WI), USA). Polymyxin B sulfate (PMB) was purchased from Molekula (Darlington, UK). Lumogen RED (LGR) and Lumogen Orange (LGO) were supplied by Kremer Pigmente (Aichstetten, Germany). Hoechst 33 528 was purchased from Thermo Fisher Scientific (USA). Cell culture materials were supplied by Biochrom GmbH (Germany) and other chemicals, reagents as well as solvents of analytical grade were obtained from commercial sources.

### Development of Dry Reverse Micelles

The reverse critical micellar concentration (rCMC) for P85, AOT, DODAB and SL in an oily mixture of medium‐chain triglycerides and mono‐ and diglycerides of caprylic acid (1:1, w/w) was determined by following the TCNQ solubilization method for oil‐soluble surfactants in nonaqueous media.^[^ ^36^ ^]^

Oily stock solutions containing 10% (w/w) of each surfactant were diluted with the oil to obtain a surfactant concentration range of 0.005% to 10%. To 0.5 mL of each dilution 0.5 mg (2.45 µmol) of TCNQ were added. After vortex mixing, the samples were vigorously shaken at 1500 rpm for 24 h on a Thermomixer C (Eppendorf, Hamburg, Germany) at 25 °C. Undissolved TCNQ was removed by centrifugation at 800 *g* for 20 min. Aliquots of 100 µL were withdrawn from the supernatant and absorbance of solubilized TCNQ was measured using a microplate reader (Tecan; Salzburg, Austria). rCMCs were determined as the inflection point of the curve obtained at a wavelength of 480 or 850 nm against the logarithm of surfactant concentrations. The plain oil was used as a reference.

Water uptake of dry reverse micelles (dRMs) was investigated according to a method previously described.^[^ ^9^ ^]^ Demineralized water was titrated in increments of 5 µg (or less) to surfactant‐in‐oil solutions described above. After addition of each aliquot of water, samples were homogenized by vortex‐mixing and incubated for 15–30 min. Water was added until a cloudy or milky appearance of the samples was observed.

Empty dRMs were prepared by dissolving surfactants in concentrations of 0.25%, 0.5%, 1%, 2.5%, 5%, and 10% (w/w) to an oily mixture consisting of medium‐chain triglycerides and mono‐ and diglycerides of caprylic acid (1:1, w/w).

Surfactant concentrations at which the formed dRMs most efficiently solubilize the model peptide drug PMB were determined. To that end, an excess amount of dry PMB powder (5 mg, 3.84 mmol) was added to 500 µL of the oily mixtures containing dRMs as described above leading to PMB containing dRMs (dRMs_PMB_).^[^ ^11^ ^]^ After incubation overnight at 25 °C while shaking at 1500 rpm on a Thermomixer C, the mixtures were centrifuged for 20 min at 800 *g* using a MiniSpin (Eppendorf). In case of complete PMB solubilization, the amount of drug was increased until saturation was reached. After centrifugation, drug concentration in saturated supernatants was determined via high‐performance liquid chromatography (HPLC).

An Elite LaChrom was utilized, consisting of a 5160 pump, a 5260 autosampler, a 5310 column oven and a 5430 diode array detector (DAD) (Hitachi, Tokyo, Japan). For all measurements, an XSelect HSS T3 column (100 mm × 4.6 mm, 3.5 µm; Waters, Dublin, Ireland) was used at 40 °C. To quantify PMB, a gradient run with a flow rate of 1 mL min^−1^ was applied using 0.1% (v/v) trifluoroacetic acid (TFA) (A) and acetonitrile + 0.1% (v/v) TFA (B) as mobile phase. After 1 min of equilibration, a gradient from 80% A to 20% A was run within 5 min followed by a 3 min backwash to the initial solvent composition. Thereafter, the system was equilibrated for 2 min. PMB was detected at the wavelength of 220 nm. For each quantification, an injection volume of 20 µL was used. According to the certificate of the supplier, PMB had a purity of 88.4% containing subtypes B1, B2, B3, and B1‐I (Figure 2A).

The declared composition was 63.8% B1, 12.3% B2, 3.2% B3, and 9.1% B1‐I (w/w). A calibration curve ranging from 0.004 to 0.5 mg mL^−1^ was established (R^2^ > 0.99) without distinguishing between subtypes. When PMB was quantified in the oily mixtures, aliquots of 10 µL were diluted 1:100 (v/v) with a mixture of water and acetonitrile (ACN) (4:6, v/v) containing 0.1% (v/v) TFA before HPLC analyses to quantify PMB as free drug.

### Characterization of Dry Reverse Micelles

In order to determine the entrapment efficacy (EE), dRMs_PMB_ were underlain with an aqueous phase of demineralized water. After 24 h of incubation at 37 °C while shaking at 300 rpm on a thermomixer, samples were centrifuged for 10 min at 9660 *g* to ensure complete phase separation. Aliquots of 50 µL were withdrawn from the aqueous phase and analyzed via HPLC as described above. EE was determined by the difference between the initial drug concentration (c) in the oily phase and the amount of free, untrapped concentration released to the aqueous phase as % drug content by using Equation (1),^[^ ^37^ ^]^

(1) $$EE=\frac{{c_{dRMs}}_{PMB}-c_{PMB\;in\;water}}{{c_{dRMs}}_{PMB}}\times 100$$

where *c_dRMs_*_*PMB*_ refers to the initial drug concentration of dRMs_PMB_ and *c*_*PMB* *in* *water*_ to the drug concentration in the aqueous phase after incubation.

Furthermore, log D values were determined using Equation (2),

(2) $$\log D=\log\left(\frac{{c_{dRMs}}_{PMB}}{c_{PMB\;in\;water}}\right)$$

*c_dRMs_*_*PMB*_ = initial drug concentration of dRMs_PMB_

*c*_*PMB* *in* *water*_ = drug concentration in the aqueous phase after incubation

### Preparation of Self‐Emulsifying Drug Delivery Systems Containing dRMs_PMB_

To prepare SEDDS‐dRMs_PMB_, 80 µL of dRMs_PMB_ were vortex mixed with 20 µL of PEG35CO. In case of SL, 70 µL of dRMs_PMB_ and 30 µL of PEG35CO was used. After homogenization, SEDDS were emulsified in demineralized water (1:100) to obtain 1% (v/v) nanoemulsions. Mean droplet size, polydispersity index (PDI) and ζ‐potential were assessed at 37 °C by dynamic light scattering (DLS) using a Zetasizer Nano ZSP (Malvern Instruments, Worcestershire, UK).

### Characterization of SEDDS‐dRMs_PMB_

The drug loading capacity in % (w/w) was calculated by Equation (3) for each formulation,^[^ ^38^ ^]^

(3) $$\begin{array}{ccc} & & \mathrm{drug}\;\mathrm{loading}\;\left[\%\left(w/w\right)\right]\\ & & \;=\frac{W\;\mathrm{of}\;\mathrm{PMB}\;\mathrm{in}\;\mathrm{SEDDS}-{\mathrm{dRMs}}_{\mathrm{PMB}}}{W\;\mathrm{of}\;\mathrm{PMB}\;\mathrm{in}\;\mathrm{SEDDS}-{\mathrm{dRMs}}_{\mathrm{PMB}}+W\;\mathrm{of}\;\mathrm{SEDDS}-{\mathrm{dRMs}}_{\mathrm{PMB}}\;\mathrm{initially}}\\ & & \;\times \;100 \end{array}$$

The weight (w) of PMB in SEDDS‐dRMs_PMB_ corresponds to the maximum payload of PMB in dRMs and the ratio of dRMs_PMB_ in SEDDS‐dRMs_PMB_. The weight of SEDDS‐dRMs_PMB_ was determined by weighing aliquots of 100 µL (*n* ≥ 3) using an analytical balance (Sartorius MSE225P‐100‐DI, Göttingen, Germany).

The rheology of SEDDS was evaluated using a cone‐plate combination rheometer (HAAKE Mars Rheometer, 40/60, Thermo Electron Karlsruhe, Germany, Rotor: C20/1) at a constant temperature of 25 °C and 52 µm gap between cone and plate. After a short incubation period at 25 °C, 150 µL of SEDDS‐dRMs_PMB_ was placed on the plate and linear shear rates ranging between 0.1 and 10 s^−1^ were applied to measure the viscosity.

The emulsification time of SEDDS‐dRMs_PMB_ was assessed by using a standard USP dissolution apparatus type II (Erweka DT 600, Heusenstamm, Germany) as described previously.^[^ ^15^ ^]^ 1 mL of SEDDS‐dRMs_PMB_ was added to 900 mL of demineralized water at 37 ± 0.5 °C, while stirring gently 50 rpm with a standard stainless‐steel paddle. The time for complete emulsification was determined visually. The transmittance of formed nanoemulsions was determined at a wavelength of 600 nm using a UV–vis spectrophotometer (Shimadzu UV mini, Korneuburg, Austria) at room temperature. Demineralized water served as 100% value.

To simulate in vivo conditions, stability tests were carried out in biorelevant media. Therefore, SEDDS were emulsified in 0.01 m HCl (pH 2) and fasted simulated intestinal fluid (FaSSIF) in a concentration of 1% (v/v). Incubated for 24 h at 37 °C at 300 rpm on a thermomixer and analyzed for changes in droplet size and PDI after 4 and 24 h by DLS. The FaSSIF medium (pH 6.5) containing bile salts and phospholipids was prepared following protocol of the supplier.^[^ ^15^ ^]^

### Drug Release Studies

Drug release was assessed by using a diffusion membrane method as previously described.^[^ ^16^ ^]^ The release of PMB from SEDDS‐dRMs_PMB_ was initially determined in demineralized water at 37 °C using dialysis tubes (Float‐A‐Lyzer G2, Spectrum Laboratories, Rancho Dominguez (CA), USA) with a cut‐off of 300 kDa. 150 µL of SEDDS‐dRMs_PMB_ was dispersed in demineralized water to a total volume of 1.5 mL in the dialysis tube and subsequently dialyzed in a 50 mL falcon tube against 15 mL of demineralized water at 37 °C. A constant temperature of 37 °C and shaking at 300 rpm were provided by a thermomixer. Aliquots of 100 µL were withdrawn from the release medium at predetermined time points and replaced by fresh release medium. The released amount of PMB was quantified via HPLC as described above. Hindrance in drug diffusion caused by the dialysis membrane was addressed by determining the release from an aqueous PMB solution serving as reference. The same procedure was carried out using 0.01 m HCl to investigate the release in the harsh gastrointestinal (GI) environment.

### Cytotoxicity Studies

The cytotoxic effect of SEDDS on a Caco‐2 cell line was evaluated via the resazurin reduction assay.^[^ ^39^ ^]^ To reach a monolayer, Caco‐2 cells were seeded on 96‐ well plates in a density of 5 × 10^5^ Caco‐2 cells·mL^−1^ per well several days before the experiment. Cells were supplemented with 10% (v/v) heat‐inactivated fetal calf serum (FCS) and a penicillin/streptomycin solution (100 units/0.1 mg·L^−1^) at 95% humidity and 37 °C in an atmosphere of 5% CO_2_ during this period. Subsequently, 100 µL of SEDDS emulsified in HEPES buffered saline (HBS) were applied to the cells in concentrations ranging from 0.01 to 1% (v/v). Pure HBS and Triton X‐100 (0.1% (v/v)) served as negative and positive control, respectively. SEDDS‐dRMs_PMB_ were removed from the cell layer after 4 h of incubation and any adherent SEDDS were washed off with 100 µL HBS. Afterward, cells were incubated for 2 h under the same conditions with 150 µL of a resazurin‐white‐MEM solution (0.1% (m/v)). Thereafter, aliquots of 100 µL of supernatants were withdrawn and their fluorescence was determined at an excitation wavelength of 540 nm and an emission wavelength of 590 nm using a microplate reader. After background extraction, cell viabilities were calculated by Equation (4),

(4) $$\mathrm{cell}\;\mathrm{viability}\;\left[\%\right]=\frac{\mathrm{Relative}\;\mathrm{fluorescence}\;{\mathrm{intensity}}_{\mathrm{sample}}}{\mathrm{Relative}\;\mathrm{fluorescence}\;{\mathrm{intensity}}_{\mathrm{positive}\;\mathrm{control}}}\times 100$$

IC_50_ values were determined using GraphPad.

### Cellular Uptake Studies

The cellular uptake of SEDDS‐dRMs_PMB_ was analyzed by flow cytometry (FC). SEDDS‐dRMs_PMB_ were labeled with 0.15% (m/m) Lumogen RED (LGR). LGR‐labeled SEDDS‐dRMs_PMB_ were dispersed and diluted in Opti‐MEM to a final non‐toxic concentration of 0.025% (m/m). In a preliminary experiment, two emission wavelength scans (excitation at 405 and 488 nm; emission from 450 to 800 nm) were performed and compared to eliminate false results due to inhomogeneous fluorescence intensities of LGR in various SEDDS‐dRMs_PMB_ concentrates. 5 days prior to the experiment, Caco‐2 cells were seeded in a 24‐ well plate (Greiner Bio‐ One, Germany) in a density of 5 × 10^4^ cells well^−1^ and cultured to obtain a monolayer. The monolayer was incubated with 500 µL of LGR‐labeled SEDDS‐dRMs_PMB_ for 4 h at 37 °C to study cellular uptake. Afterward, cells were detached by adding 200 µL of Accutase and washed with 500 µL of ice cooled 10 mm PBS pH 7.4 thrice. The amount of LGR loaded SEDDS‐dRMs_PMB_ uptaken by Caco‐2 cells was quantified via FC (Attune NxT Flowcytometer, Thermofisher Scientific). A custom‐written MatLab program using a neuronal network for autonomous gating was applied for data analysis. The relative mean fluorescence intensity (RMFI) values represent the average concentration of LGR‐labeled SEDDS taken up per cell and were calculated by Equation (5),

(5) $$\mathrm{RMFI}=\frac{\mathrm{MFI}\;\left(S\right)}{\mathrm{MFI}\;\left(C\right)}-1$$

where MFI (S) refers to the mean fluorescence intensity of the treated sample and MFI (C) to the mean fluorescence intensity of the control.

Accordingly, the RMFI value of untreated Caco‐2 cells is always 0. 10 000 cells were analyzed at constant gating settings for each measurement. Deceptive effects of logarithmic scaling for low signals and compensated data were avoided by using the logicle' display method. The y‐axis (count) was scaled with respect to the histogram maximum in which the black dashed line defines the fluorescence intensity threshold (<0.3% cells of control are above). All cells displaying fluorescence intensities above this threshold were counted into percentage of uptake.

Confocal laser scanning microscopy (Leica TCS SP8) was conducted to confirm cellular uptake and to evaluate the intracellular distribution of SEDDS‐dRMs_PMB_ in Caco‐2 cells. An 8‐ well chamber slide (µ‐slide, Ibidi) was prepared with a cell density of 1·10^5^ Caco‐2 cells well^−1^ and cultured as described above. Afterward, 300 µL of LGR‐labeled SEDDS‐dRMs_PMB_ were prepared as described above, added to the cells and incubated for 4 h. After rinsing Caco‐2 monolayers with pre‐warmed 10 mm PBS pH 7.4 twice, Hoechst 33528 (1 µg·mL^−1^) was applied on the cells for 8 min to stain nuclei. Fluorescence images of each well were recorded using equal confocal settings. ImageJ was used for postprocessing and analysis. Three xy‐images of an image stack taken at 0.2 µm z‐step length were used to prepare the yz‐ and xz‐projections. The intensity distribution along the z‐axis of the cell layer was calculated based on the raw data of each SEDDS‐dRMs_PMB_. The resulting x‐y images display a maximum‐z‐projection located around nucleus (d_nuc_ = 0 µm), that is, the average maximum fluorescence intensity of the nucleus, which corresponds approximately to the middle of the cell layer along z‐direction.

A sliding window approach written in MatLab was used to determine the intensity‐distribution of each formulation across the Caco‐2‐layer. Briefly, the mean intensity distribution along the z‐axis of the stack image data was determined by using a volume of the size W/a × H/a × z, (tested for a = 1, 9, 36). The cell‐layer boundaries (top, bottom) within the volume were obtained in a semiautomatic approach (based on the nucleus and SEDDS‐dRMs_PMB_‐intensity profile). The program proposes the cell‐ layer‐boundaries which is subsequently verified by the user via the image data by using a maximums‐projection of selected volume in y‐direction. Based on the volume‐fraction curves for each sample at three different locations (−3 µm, cell membrane proximal; +3 µm bottom proximity; 0 µm, nucleus of cell) within the well, an average curve of the MFI/plane was calculated for each SEDDS‐ dRMs_PMB_. From the recorded image‐data, the colocalization between uptaken SEDDS‐dRMs_PMB_ and the nucleus was determined using the ObjectFinder‐application in MatLab. A minimum of a 50% overlap of SEDDS‐dRMs_PMB_‐objects with the nucleus‐object was counted as colocalized.

### Membrane Interaction – Endosomal Escape Studies

The membrane interaction of SEDDS‐dRMs_PMB_ were determined on red blood cells (RBC) under physiological conditions in‐vitro as it is widely used to mimic the in vivo conditions after endosomal internalization of nanocarriers.^[^ ^27^ ^]^ The RBC were kindly donated by Tirol Kliniken (Innsbruck, Austria).

SEDDS‐dRMs_PMB_ emulsified in sterile glucose HBS pH 7.4 in concentrations ranging from 0.001% to 1% were added to equal volumes of erythrocyte:HBS suspensions (1:100, v/v) and incubated for 4 h at 37 °C under shaking 300 rpm on a thermomixer. As positive and negative controls served Triton X‐100 in HBS (2% (v/v)) and pure HBS, respectively. To separate non‐hemolyzed erythrocytes, the samples were subsequently centrifuged for 10 min at 500 *g* utilizing a MiniSpin. The extent of hemolysis was determined by measuring the hemoglobin absorbance in supernatants at a wavelength of 415 nm using a microplate reader. The hemolysis rate was calculated using Equation 6,

(6) $$\mathrm{Hemolysis}\;\left[\%\right]=\frac{\mathrm{Abs}\left(T\right)-\mathrm{Abs}\left(\mathrm{neg}\right)}{\mathrm{Abs}\left(\mathrm{pos}\right)-\mathrm{Abs}\left(\mathrm{neg}\right)}\times 100$$

Where Abs (T) refers to the absorbance of test sample, Abs (neg) to the absorbance of the negative and Abs (pos) to the absorbance of the positive control. To determine the half‐maximal hemolytic concentration (HC_50_) the hemolysis rate was plotted against the log concentration of each SEDDS‐dRMs_PMB_ using GraphPad. In addition, colocalization of lysosomes and SEDDS were studied via confocal microscopy as described above with some minor modifications. SEDDS‐dRMs_PMB_ were labeled with Lumogen Orange (LGO) in a concentration of 0.15% (m/m). For lysosomal staining, the cells were incubated for 30 min with LysoView 633 (1 mg mL^−1^) instead of using Hoechst 33528.

### Antimicrobial Activity Studies

The antimicrobial activity of SEDDS‐dRMs_PMB_ was evaluated by determining the minimum inhibitory concentration (MIC) following a slightly modified method previously described.^[^ ^40^ ^]^ In brief, several single strains of *E. coli* DH5α were transferred into falcon tubes containing 3 mL of sterile Müller Hinton Broth. Bacteria were grown at 37 °C overnight while shaking at 150 rpm using an orbital shaker‐incubator (ES‐80, Grant‐Bio, Cambridgeshire, UK). Bacterial suspensions with a turbidity equivalent to the McFarland PMS 0.5 standard were prepared in sterile water and diluted 1:300 with sterile Müller Hinton Broth to achieve 5 × 10^5^ colony‐forming units. MIC was determined via the broth microdilution method. Briefly, serial dilutions of either an aqueous PMB solution or SEDDS‐dRMs_PMB_, ranging from 5 to 0.039 µg mL^−1^ PMB, or of blank SEDDS‐dRMs as control were prepared and added to the bacterial suspension in a 96‐well plate. Sterile culture medium diluted with water and sterile water with bacterial suspension were used as positive and negative control, respectively. After 20 h of incubation at 35 ± 2 °C the absorbance was measured at a wavelength of 600 nm using a microplate reader. MICs were determined as the lowest concentration inhibiting the microbial activity.

### Statistical Data Analysis

Statistical data analyses were carried out using Student's *t*‐test to determine the significant difference between two means on the assumption of unequal variance. The level of *p* ≤ 0.05 was significant, *p* ≤ 0.01 very significant and *p* ≤ 0.001 highly significant. Results were reported as the mean of at least three replicates ± standard deviation (SD).

## Conflict of Interest

The authors declare no conflict of interest.

## Supporting information

Supporting Information

## Data Availability

The data that support the findings of this study are available from the corresponding author upon reasonable request.
