# Supplementary material for: Self‐Emulsifying Drug Delivery Systems (SEDDS) Containing Reverse Micelles: Advanced Oral Formulations for Therapeutic Peptides
Source: Adv Healthc Mater. 2023 Sep 20;12(31):2302034. doi: 10.1002/adhm.202302034 (PMC11468804; doi:10.1002/adhm.202302034)
Supplement: Supplementary file 1 — Supporting Information [file ADHM-12-2302034-s001.pdf]

ADVANCED HEALTHCARE MATERIALS

Supporting Information

for *Adv. Healthcare Mater.*, DOI 10.1002/adhm.202302034

Self-Emulsifying Drug Delivery Systems (SEDDS) Containing Reverse Micelles: Advanced Oral Formulations for Therapeutic Peptides

*Arne Matteo Jörgensen, Christian Steinbring, Daniel Stengel, Dennis To, Pascal Schmid and Andreas Bernkop-Schnürch**

Self-emulsifying drug delivery systems (SEDDS) containing reverse micelles: Advanced oral formulations for therapeutic peptides

*Arne Matteo Jörgensen[1], Christian Steinbring[1], Daniel Stengel[1], Dennis To[1], Pascal Schmid[1], Andreas Bernkop-Schnürch[1]**

[1] Department of Pharmaceutical Technology, University of Innsbruck, Institute of Pharmacy, Center for Chemistry and Biomedicine, Innrain 80-82, 6020 Innsbruck, Austria

---

*Corresponding author
[1] Department of Pharmaceutical Technology, University of Innsbruck, Institute of Pharmacy, Center for Chemistry and Biomedicine, 6020 Innsbruck, Austria
Tel.: +43 512 507 58 600
Fax: +43 512 507-58699
Email: Andreas.Bernkop@uibk.ac.at

Abstract:

Alternative methods to hydrophobic ion pairing for the formation of lipophilic complexes of peptide drugs to incorporate them in lipid-based nanocarriers such as self-emulsifying drug delivery systems (SEDDS) for oral administration are highly on demand. Such an alternative might be reverse micelles.

Within this study, SEDDS containing dry reverse micelles (dRMs$_{PMB}$) formed with an anionic (sodium docusate; AOT), cationic (dimethyl-dioctadecyl-ammonium bromide*;* DODAB), amphoteric (soy lecithin; SL) or non-ionic (polysorbate 85; P85) surfactant loaded with the model peptide drug polymyxin B (PMB) were developed. They were characterized regarding size, payload, release kinetics, cellular uptake and peptide activity.

SEDDS exhibited sizes from 22.2 ± 1.7 nm (AOT-SEDDS-dRMs$_{PMB}$) to 61.7 ± 3.2 nm (P85-SEDDS-dRMs$_{PMB}$) with payloads up to 2% that were ~7-fold higher than those obtained via hydrophobic ion pairing. Within 6 h P85-SEDDS-dRMs$_{PMB}$ and AOT-SEDDS-dRMs$_{PMB}$ showed no release of PMB in aqueous medium, whereas DODAB-SEDDS-dRMs$_{PMB}$ and SL-SEDDS-dRMs$_{PMB}$ showed a sustained release. DODAB-SEDDS-dRMs$_{PMB}$ improved uptake by Caco-2 cells most efficiently reaching even ~100% within 4 h followed by AOT-SEDDS-dRMs$_{PMB}$ with ~20% and P85-/SL-SEDDS-dRMs$_{PMB}$ with ~5%. The peptide drug maintained its antimicrobial activity in all SEDDS-dRMs$_{PMB}$.

According to these results, SEDDS containing dRMs might be a game changing strategy for oral peptide drug delivery.

Keywords: Nanocarriers, Oral peptide delivery, Polymyxin B, Drug release, Payload enhancement

Figure S-1:

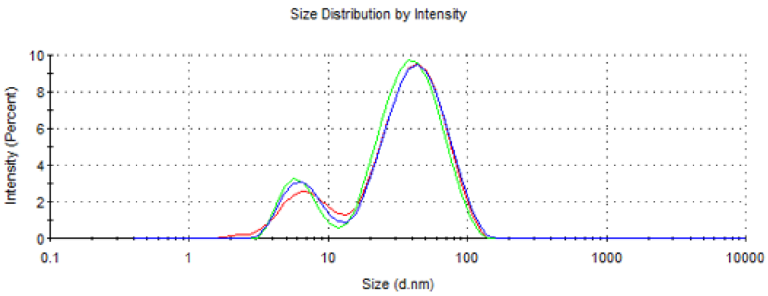

Figure S-1: Size distribution by intensity of AOT-SEDDS-dRMs$_{PMB}$

Figure S-2:

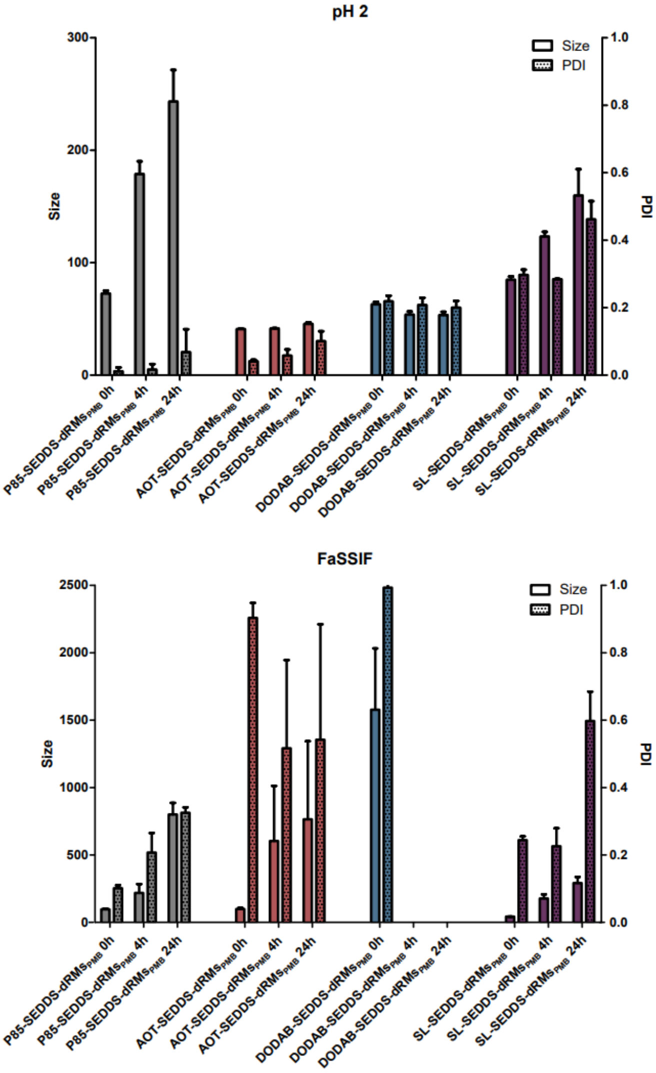

Figure S-2: Size and PDI of P85- (grey), AOT- (red), DODAB- (blue) and SL-SEDDS-dRMs$_{PMB}$ (violet) dispersed in 0.01 M HCl (pH 2) and FaSSIF.

Figure S-3:

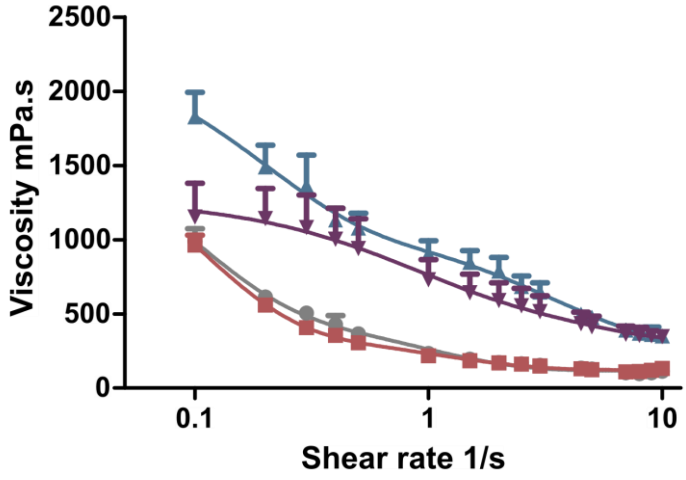

Figure S-3: Viscosity of P85- (grey ●), AOT- (red ■), DODAB- (blue ▲) and SL-SEDDS-dRMs$_{PMB}$ preconcentrates (violet ▼).

Table S-1: dRM$_s$-SEDDS as controls for antimicrobial activity.

	Absorbance at 600 nm wavelength			
PMB concentration [µg·mL^{-1}]	P85-SEDDS-dRMs	AOT-SEDDS-dRMs	DODAB-SEDDS-dRMs	SL-SEDDS-dRMS
5	0.342 ± 0.021	0.364 ± 0.008	0.388 ± 0.088	0.444 ± 0.011
2.5	0.283 ± 0.013	0.342 ± 0.014	0.262 ± 0.062	0.374 ± 0.028
1.25	0.269 ± 0.024	0.296 ± 0.006	0.284 ± 0.022	0.302 ± 0.010
0.625	0.279 ± 0.019	0.332 ± 0.026	0.284 ± 0.004	0.313 ± 0.010
0.3125	0.258 ± 0.021	0.270 ± 0.005	0.250 ± 0.012	0.261 ± 0.024
0.15625	0.276 ± 0.014	0.254 ± 0.016	0.266 ± 0.024	0.260 ± 0.039
0.078125	0.256 ± 0.007	0.247 ± 0.008	0.243 ± 0.018	0.255 ± 0.002
0.0390625	0.271 ± 0.028	0.2627 ± 0.016	0.261 0.042	0.273 ± 0.018
